# Single Metal Atoms on Oxide Surfaces: Assessing the Chemical Bond through ^17^O Electron Paramagnetic Resonance

**DOI:** 10.1021/acs.accounts.2c00606

**Published:** 2022-11-28

**Authors:** Enrico Salvadori, Paolo Cleto Bruzzese, Elio Giamello, Mario Chiesa

**Affiliations:** † Department of Chemistry and NIS Centre of Excellence, University of Turin, via Giuria 9, 10125 Torino, Italy; ‡ Felix Bloch Institute for Solid State Physics, Leipzig University, Linnéstr. 5, 04103 Leipzig, Germany

## Abstract

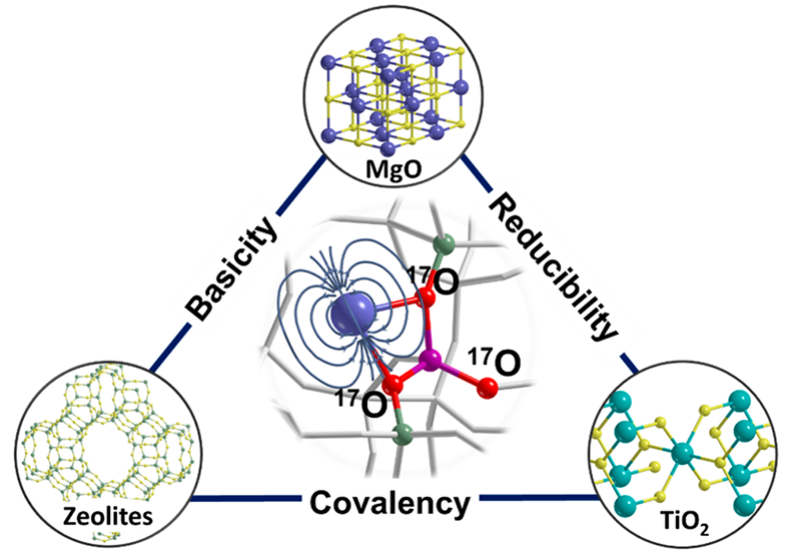

Even in the gas phase single atoms possess catalytic properties, which can be crucially enhanced and modulated by the chemical interaction with a solid support. This effect, known as electronic metal–support interaction, encompasses charge transfer, orbital overlap, coordination structure, etc., in other words, all the crucial features of the chemical bond. These very features are the object of this Account, with specific reference to open-shell (paramagnetic) single metal atoms or ions on oxide supports. Such atomically dispersed species are part of the emerging class of heterogeneous catalysts known as single-atom catalysts (SACs). In these materials, atomic dispersion ensures maximum atom utilization and uniform active sites, whereby the nature of the chemical interaction between the metal and the oxide surface modulates the catalytic activity of the metal active site by tuning the energy of the frontier orbitals. A comprehensive set of examples includes fourth period metal atoms and ions in zeolites on insulating (e.g., MgO) or reducible (e.g., TiO_2_) oxides and are among the most relevant catalysts for a wealth of key processes of industrial and environmental relevance, from the abatement of NO_*x*_ to the selective oxidation of hydrocarbons and the conversion of methane to methanol.

There exist several spectroscopic techniques able to inform on the geometric and electronic structure of isolated single metal ion sites, but either they yield information averaged over the bulk or they lack description of the intimate features of chemical bonding, which include covalency, ionicity, electron and spin delocalization. All of these can be recovered at once by measuring the magnetic interactions between open-shell metals and the surrounding nuclei with Electron Paramagnetic Resonance (EPR) spectroscopy. In the case of oxides, this entails the synthesis of ^17^O isotopically enriched materials. We have established ^17^O EPR as a unique source of information about the local binding environment around oxygen of magnetic atoms or ions on different oxidic supports to rationalize structure–property relationships. Here, we will describe strategies for ^17^O surface enrichments and approaches to monitor the state of charge and spin delocalization of atoms or ions from K to Zn dispersed on oxide surfaces characterized by different chemical properties (i.e., basicity or reducibility). Emphasis is placed on chemical insight at the atomic-scale level achieved by ^17^O EPR, which is a crucial step in understanding the structure–property relationships of single metal atom catalysts and in enabling efficient design of future materials for a range of end uses.

## Key References

- ChiesaM.; GiamelloE.; Di ValentinC.; PacchioniG.; SojkaZ.; Van DoorslaerS.Nature of the Chemical Bond between Metal Atoms and Oxide Surfaces: New Evidences from Spin Density Studies of K Atoms on Alkaline Earth Oxides. J. Am. Chem. Soc.2005, 127, 16935–169441631623910.1021/ja0542901.^1^*Here by means of ^17^O EPR and DFT, we showed that single K atoms are relatively strongly bound to oxide anions at particular morphological irregularities of the surface and provide clear insights into the nature of the metal–support interaction.*
- LivraghiS.; ChiesaM.; PaganiniM. C.; GiamelloE.On the Nature of Reduced States in Titanium Dioxide As Monitored by Electron Paramagnetic Resonance. I: The Anatase Case. J. Phys. Chem. C2011, 115, 25413–25421.^2^*The introduction of the ^17^O magnetic isotope at the surface and in the bulk of TiO_2_ allows us to elucidate the nature of TiO_2_ reduced states originated in various ways, providing evidence for the presence of delocalized states in the anatase polymorph.*
- MorraE.; SignorileM.; SalvadoriE.; BordigaS.; GiamelloE.; ChiesaM.Nature and Topology of Metal–Oxygen Binding Sites in Zeolite Materials: ^17^O High-Resolution EPR Spectroscopy of Metal-Loaded ZSM-5. Angew. Chem., Int. Ed.2019, 58, 12398–1240310.1002/anie.20190648831294524.^3^*In this work through a combination of selective ^17^O isotopic enrichment and the unique properties of open-shell s-state monovalent group 12 cations, we derive a site-specific topological description of active sites in an MFI zeolite.*
- BruzzeseP. C.; SalvadoriE.; JägerS.; HartmannM.; CivalleriB.; PöpplA.; ChiesaM.^17^O-EPR Determination of the Structure and Dynamics of Copper Single-Metal Sites in Zeolites. Nat. Commun.2021, 12, 46383433091410.1038/s41467-021-24935-7PMC8324863.^4^*Here, ^17^O EPR is combined with DFT modeling to determine the local structure of single site Cu^II^ species, quantify the covalency of the metal–framework bond and assess how this scenario is modified by the presence of solvating water molecules.*

## 1 Introduction

“Adsorbed, chemisorbed, embedded, anchored, grafted” are all different words used to describe the variety of bonding interactions of an atom with the surface of a support.^5^ Spatially isolated metal atoms on surfaces represent a relevant class of heterogeneous catalysts, referred to as “single atom catalysts” (SACs). Here “single atom” denotes well isolated and atomically dispersed species at specific surface sites. The basic rules of chemistry apply to this interfacial situation, which define the interaction between the atom and the surface as dispersive, covalent or ionic depending on the degree of orbital overlap and energy difference of the interacting orbitals. The understanding of such interactions grew up in parallel with the development of methods for the controlled deposition of metal particles and, in general, with the improvement of the performances of advanced surface science^6,7^ and computational techniques.^8,9^ Evolving from early models,^10−12^ the evidence of an enhancement of the catalytic activity induced by electronic interactions between a metal and an oxide^13^ led to the definition in 2012 of the electronic metal–support interaction (EMSI).^14^ EMSI encompasses the metal–oxide interactions based on charge transfer, orbital overlap etc. In other words, the crucial features of the chemical bond, whose formation is critically dependent on the spin state of the electrons.

For open-shell metals, the spin, and its delocalization, is central in dictating design principles for the development of new sustainable catalytic pathways^15^ and plays a key role in the evolution of new materials^16^ and quantum information technologies.^17^

**Figure 1:**
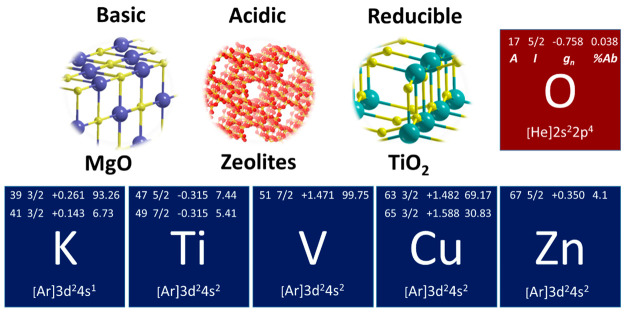
Single metal atoms and relative oxide supports described in this Account. For each element, including oxygen, the relevant magnetic properties are listed: isotope (*A*), nuclear spin (*I*), nuclear *g* factor (*g*_*n*_), and natural abundance (%Ab).

A powerful yet underexploited experimental technique is Electron Paramagnetic Resonance (EPR) because of its intrinsic ability to monitor the electron spin density in paramagnetic systems.^18,19^ EPR has been successfully applied to study ensembles of single atoms dispersed on polycrystalline surfaces–the object of this Account–and on thin oxide films.^20^ Noteworthy, the recent combination of EPR with scanning tunneling microscopy (STM) enabled addressing the EPR signature of *an individual atom* on the surface.^21−23^ In the case of metals on oxide surfaces, the coupling between the electron and ^17^O nuclear spins is a unique source of information about the local binding environment around the open-shell metal center that allows one to rationalize structure–property relationships in the most diverse systems.^24−31^ In our laboratory ^17^O EPR has been used over the past 15 years^1−4,32−38^ to investigate the interaction of single paramagnetic metal atoms or ions with different oxidic supports in order to monitor the redistribution of the electron spin density and to understand, in this way, the specific features of the metal–oxide chemical interaction. Here we will focus on single atoms and ions with complementary valence electron configurations across the fourth period of the periodic table, from K to Zn, and their interactions with oxide substrates featuring complementary chemical properties (Figure 1). For simplicity, regardless of whether they are neutral or charged, we will address them as single atoms. Emphasis will therefore be on studies from our group focused on oxide surfaces. For details on other supports, synthetic procedures and catalytic performances of SACs, the interested reader is referred to the literature.^5,39−43^

## 2 ^17^O Hyperfine Spectroscopy

Single metal species interact with oxide surfaces forming new bonds at the interface, which can range from weak interactions, dominated by dispersive forces and polarization effects, to covalent bonds, involving the mixing of metal and oxide orbitals, up to net electron transfer interactions resulting in ionic bonds. All these situations, which crucially depend on the nature of the oxide support, can be monitored for paramagnetic atoms by assessing the total spin density distribution, i.e., the total electron density of electrons of one spin (α) minus the total electron density of the electrons of the opposite spin (β). The spin density is a property directly related to the hyperfine interaction and associated coupling constants, which are experimental observables in a EPR measurement.^44^ The hyperfine interaction splits the EPR resonance transition in 2*nI* + 1 hyperfine lines, where *n* is the number of equivalent nuclei and *I* is the nuclear spin quantum number. Therefore, the number of hyperfine lines in the EPR spectrum directly identifies the presence of a single metal species (*n* = 1) through the detection of 2*I* + 1 transitions. The integrated intensity of a Continuous Wave (CW) EPR spectrum is directly proportional to the concentration of spin centers in the sample. In our case, if the total metal loading is known (e.g., via ICP analysis) then the fraction of EPR active centers can be determined.^4^ The splitting between the 2*I* + 1 transitions–referred to as the hyperfine coupling (hfc) constant–directly reflects the perturbation of the metal wave function induced by the substrate, i.e., the nature of the chemical bond. Magnetic interactions are very sensitive to the chemical properties of the binding site and are able to probe the full range of chemical interactions. However, to draw meaningful conclusions and avoid being misled by hasty interpretations, hfc to *all* nuclei (the single atom and the ligands) encompassed by the unpaired electron wave function must be measured. Notice that hfc in general consists of a dipolar (orientationally dependent) and an isotropic term; therefore, in the case of polycrystalline materials, where a “powder” average of all orientations with respect to the magnetic field is observed, the EPR spectra can be rather complex and simulation analysis is needed to reliably extract the relevant parameters. For details on the analysis of powder EPR spectra, the interested Reader is referred to the many books and review articles available in the literature.^18,19,44^ Indeed, it is the analysis of all the hyperfine data that allows the determination of the atomistic and electronic structure of coordinated metal atoms. In more detail, the isotropic hfc, *a*_iso_, is a direct measure of the s electron spin density, ρ_N_^α–β^ at a given nucleus N, whereas the dipolar hfc, *T*, yields the contribution of orbitals with higher angular momentum (p, d, etc.).^44^ The isotropic hyperfine coupling at a certain nucleus N is given in energy units by
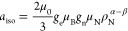
1where μ_0_ is the vacuum permeability, *g*_e_ the electron *g*-factor, μ_B_ the Bohr magneton, *g*_n_ the nuclear *g* factor, and μ_N_ the nuclear magneton. With reference to ^17^O, knowing *a*_iso_ and *T* for the atomic species, and assuming that the hfc at a given nucleus is proportional to the electron spin density at that nucleus, it is possible to estimate the spin population in s-type orbitals (ρ_s_) and p-type orbitals (ρ_p_). For an unpaired electron (free electron, *g*_e_ = 2.0023) on a ^17^O nucleus with a unitary spin population (ρ_s_ = 1) in an s-type orbital, one would observe an isotropic hyperfine coupling constant of *a*_0_ = −4622.83 MHz. If the electron resides in a p-type orbital, one would observe a uniaxial hyperfine constant of *b*_0_ = 130.5 MHz. Including a correction for the difference in the *g* values, the spin populations in s-type and p-type orbitals can thus be estimated as
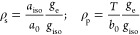
2For instance, for the case relevant to this Account–a single metal atom on an oxide surface–the ratio of the measured isotropic and dipolar hfc to the corresponding values for the free atomic state quantifies the contribution of the metal atomic orbitals to the molecular orbital containing the unpaired electron. On the other hand, the hfc with ^17^O nuclei reflects the electron spin density distribution over the coordinating ligands, which provide a clear-cut answer to the thorny question on how metals bind on oxide surfaces.

Experimentally, hfc can be directly detected in a standard CW-EPR experiment if large enough. If small, they can be recovered by means of hyperfine techniques such as Electron Nuclear Double Resonance (ENDOR), Electron Spin Echo Envelope Modulation (ESEEM) and Hyperfine Sublevel Correlation (HYSCORE) spectroscopies,^45,46^ which exploit the interaction between electron and nuclear magnetic moments to measure the NMR spectrum associated with the paramagnetic center. ENDOR experiments (in both CW or pulse variants) are based on a combination of micro- and radiowaves to measure the response of the electron spin as an incident radio frequency (rf) sweeps through different nuclear transitions.^45^ ESEEM and HYSCORE are based on a sequence of microwave (mw) pulses to generate an electron spin echo, whose intensity is monitored as a function of the variation of one or more time intervals between the pulses. The resulting time-domain signal is modulated by the nuclear frequencies, and after Fourier transformation a frequency-domain spectrum reproducing the nuclear frequencies is obtained.^46,47^

In general, the two classes of hyperfine techniques give complementary information. ESEEM techniques are mainly used to detect small hfc (<5 MHz), while ENDOR techniques are usually preferred to observe larger nuclear frequencies, i.e., larger hfc.

## 3 ^17^O Surface Doping of Metal Oxides

Hyperfine techniques require the presence of magnetic nuclei (i.e., nuclei having a spin). In the case of oxides this implies magnetic oxygen ions. Oxygen has only one magnetic isotope, ^17^O, characterized by a high spin quantum number (*I* = 5/2) and a natural abundance (0.038%), far too low to detect any hyperfine structure in naturally occurring samples. The exploitation of hyperfine techniques to assess the metal–oxygen bond requires therefore isotopic enrichment of the oxide matrix. This involves cost and effort but can be very rewarding. In the case of polycrystalline materials, the high cost of ^17^O isotopically enriched reagents makes bulk synthesis very inconvenient. Moreover, opposite to ^17^O NMR studies,^48^ where uniform substitution of ^17^O throughout the lattice is often sought to allow for quantitative measurements, selective isotopic enrichment can be advantageous in the case of EPR studies of single atoms on the surface. ^17^O surface enrichment can be performed following specific procedures that depend on the nature of the oxide. For nonreducible oxides (such as alkali-earth metal oxides, alumina, zeolites, etc.) the preferred isotope carrier is isotopically labeled water (H_2_^17^O).^1,33−35^ In this case hydration/dehydration cycles using H_2_^17^O vapors provide an effective and atom-efficient method to incorporate ^17^O isotopes at the surface. By adjustment of contact time and temperature, the process can be selective, limiting the isotopic exchange to the most reactive surface sites, which are the very sites involved in the stabilization of surface single atoms.

**Figure 2:**
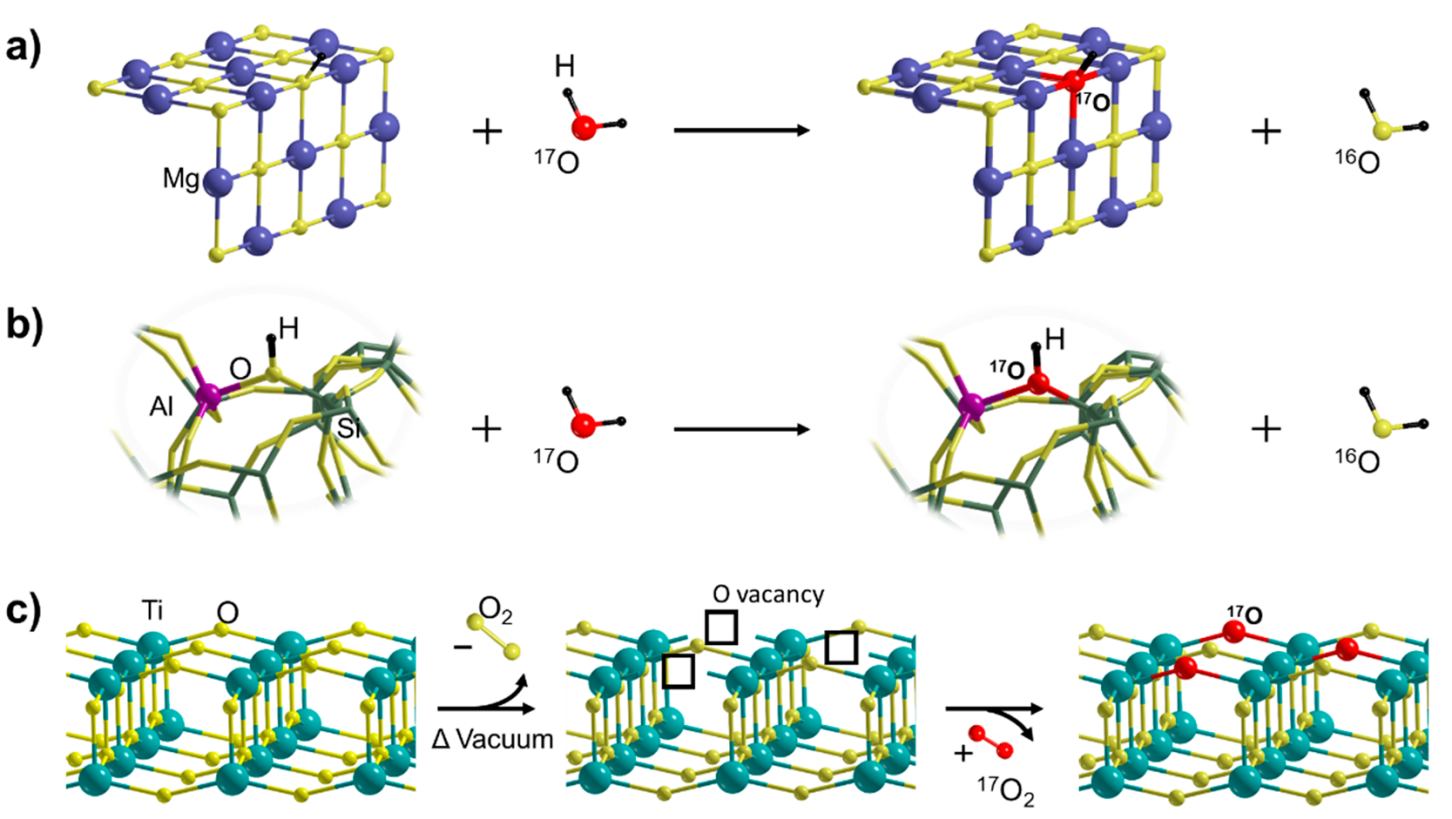
^17^O enrichment of different oxide systems: (a) alkaline earth oxides, (b) zeolites, (c) TiO_2_.

In the case of rock-salt structures such as MgO (Figure 2a), the exchangeable and chemically relevant sites correspond to three- and four-coordinated oxygen ions at corners and edges, whereas five-coordinated sites at dominant (100) faces are less involved in the exchange process.^33^ Similarly, zeolite (Figure 2b) can readily exchange their Si–O–Al and Si–O–Si framework sites with the oxygen of H_2_^17^O, while keeping their highly crystalline frameworks.^3,4,37,48,49^ The reactivity order is Si–O–Al > Si–O–Si, where Si–O–Al are the privileged metal binding sites because of charge compensation. The reported isotopic enrichment protocols were carefully scrutinized against structural or morphological alteration of the pristine oxide materials. Such careful structural characterization should always be performed when water is employed as isotopic carrier in light of possible oxide sintering or Al leaching from the zeolite framework.^3^

For reducible oxides such as TiO_2_ (Figure 2c), the strategy exploits the easy lattice oxygen depletion at the surface achieved by thermal treatments under vacuum.^2^ This generates oxygen vacancies, which can be replenished by heating in ^17^O_2_ atmosphere.^50^ Due to the surface nature of oxygen vacancies, this method ensures a selective isotopic enrichment of the surface.^51^

## 4 The Effect of Acid–Base Properties of the Support: Single Metal Atoms and Ions with 4s^1^ Electron Configuration

**Figure 3:**
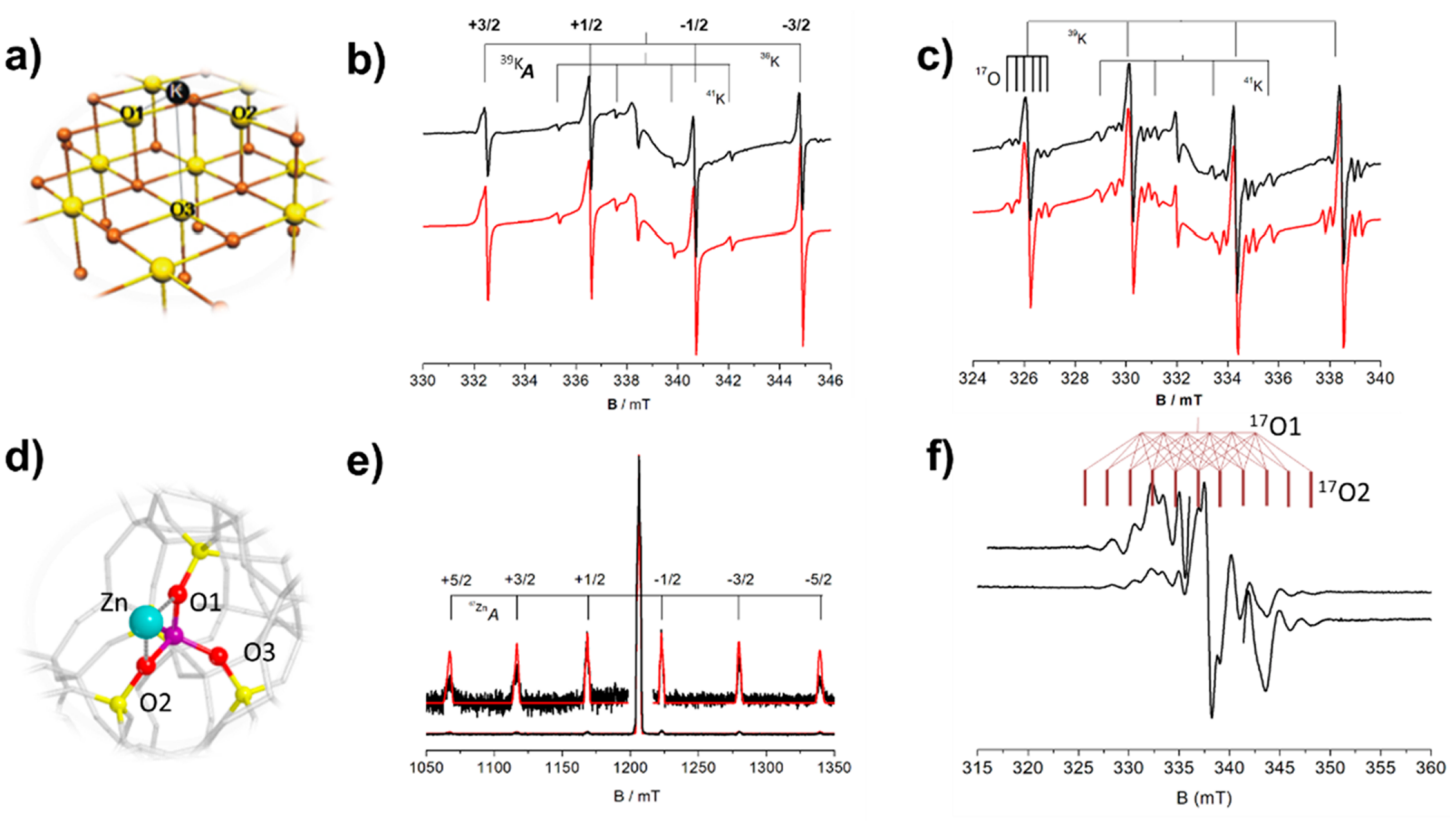
(a) Model for single K atoms adsorbed on MgO. (b) CW-EPR spectrum of single K atoms on Mg^16^O and (c) on surface enriched Mg^17^O. (d) Model for single Zn^+^ ions adsorbed on H-ZSM-5. (e) Pulsed EPR spectrum of single Zn^+^ ions on H-ZSM-5. (f) CW-EPR spectrum of Zn^+^ ions on ^17^O enriched H-ZSM-5. Black lines, experimental; red lines, simulations. Panels a, b, and c reproduced with permission from ref (1). Copyright 2005 American Chemical Society. Panels e and f adapted with permission from ref (3). Copyright 2019 Wiley.

Central to the problem of the metal atom–oxide interaction is the ability to monitor the electron spin density distribution over the atoms of the support. While the number of EPR lines and their relative intensity provides direct compelling evidence for the presence of single metal atoms on the surface, the hfc with ligated atoms of the support encodes important information on the electronic structure and binding geometry. A paradigmatic example is that of neutral alkali metal atoms stabilized on the surface of alkaline earth oxides,^1,52,53^ which represent an excellent test-bed to interpret the bonding mechanisms of neutral metals on nonreducible oxides.^20^ In the following we will discuss the case of K on MgO (K/MgO, Figure 3a).

The four hyperfine lines observed in a standard CW-EPR spectrum (Figure 3b) are due to the interaction of the unpaired electron with the *I* = 3/2 nuclear spin of K and firmly demonstrate the presence of single potassium atoms on the surface of MgO.^1^ The EPR spectrum of Figure 3b proves that surface K species retain the recognizable parentage of alkali atoms in the gas phase, but are subject to large perturbations arising from strong atom–surface interactions. In fact, the isotropic hfc of the metal is reduced by about 50% as compared to that of gas phase K atoms.^54^ A naive interpretation may point to a partial charge transfer from the metal to the surface. However, analysis of the complementary ^17^O hfc in an enriched Mg^17^O sample showed that K atoms bind strongly to two surface oxygen ions (O1 and O2 in Figure 3a) and weakly to a third (O3 in Figure 3a), but the measured maximum ^17^O hfc of ≈9 MHz is far too small to account for a significant spin delocalization over the matrix ions.^1^ This evidence thus firmly excludes that the origin of the lowered hfc observed at the K nucleus is due to a spin (charge) delocalization over the oxide support. In fact, the mechanism responsible for the reduction of the spin density at the K nucleus^1^ is principally a polarization of the singly occupied 4s orbital of neutral K atoms induced by Pauli repulsion effects brought about by the lone pairs of surface oxygens. The net result of this interaction is to lift in energy the 4s K orbital, favoring some degree of sp hybridization. This “expanded atom” (or polarized) state explains the reduced hfc with respect to the free atom without invoking any significant metal to surface electron transfer. This is a general bonding scheme for neutral metals on the surface of basic oxides^20^ and depends on the degree of interaction between the oxide ion lone pairs and the *n*s orbital or, in other words, on the basicity of the oxide. A systematic study of alkaline earth oxides demonstrated that the reduction of the metal hfc linearly correlates with the well-known trend of basic strength MgO < CaO < SrO < BaO.^52^

To fully appreciate the role of the substrate, it is instructive to compare the data on MgO to a different oxide substrate featuring opposite characteristics, i.e., a covalent and acidic nature, namely, zeolite (Figure 3d). Zeolites are composed of corner sharing SiO_4_ and AlO_4_ tetrahedra, arranged into three-dimensional frameworks in such a manner that they contain regular channels and cavities of molecular dimensions. The substitution of aluminum (formally Al^3+^), in place of silicon (Si^4+^), produces a net negative charge, which is balanced by acidic (Brønsted) protons (or other cations) resident in the cavities. When incursive atoms from the vapor of, for example, alkali metals enter a dehydrated zeolite, they are spontaneously ionized reducing the acidic protons and forming a variety of unusual ions, clusters, and filamentary structures.^55^ Particularly interesting to our discussion is the case of zinc. The exposure of a protonated zeolite to Zn vapors leads to the reduction of H^+^ Brønsted sites and the formation of the unusual monovalent Zn^+^ species, characterized by a 4s^1^ electronic structure, which are isoelectronic with K.^56^ The EPR spectrum of sublimated Zn in zeolites is characterized by a nearly isotropic signal flanked by six evenly spaced satellite transitions due to the hfc with ^67^Zn (*I* = 5/2, 4.1% natural abundance).

While the six satellite transitions (Figure 3e) provide evidence for the presence of single metal ions, analysis of the ^67^Zn^+^ hfc points to 80% electron spin density localization in the 4s Zn orbital, proving the formation of a genuine Zn^+^ ion. The 20% missing spin density is shared between two equivalent coordinating oxygen atoms as testified by the 11 hyperfine lines (hfc ≈ 60 MHz) of the ^17^O EPR spectrum (Figure 3f). While the local metal coordination is similar for K/MgO and Zn/ZSM-5, the electronic structure is dramatically different. The coordinating oxygen ions of the ionic and basic MgO strongly polarize the K atom wave function with a minute spin delocalization to the surface (^17^O hfc ≈ 9 MHz); on the other hand the more covalent and acidic zeolite yields a bond mostly ionic, with a non-negligible degree of covalency (^17^O hfc ≈ 60 MHz). This comparison illustrates the unique level of details provided by ^17^O hfc in the description of the metal coordination environment in disordered systems. This knowledge allows us to pinpoint the structure of the metal binding sites with atomistic precision and discriminate among different potential binding sites and it is the prerequisite to map the spatial distribution of single metal atoms in the nanometer range.^57^ When the ^17^O hfc is large enough to be resolved, the CW-EPR spectrum provides a handle to assess the level of isotopic enrichment at the metal site. In the case of K/MgO and Zn/ZSM-5, both characterized by a digonal coordination, this is done by comparing the relative intensity of the ^17^O hfc pattern and assuming a binomial distribution. In this way, we estimated a 10% ^17^O enrichment for K/MgO^1^ and a 70% enrichment for Zn/ZSM-5.^3^ Considering the procedure described in section 3, we note that the isotopic enrichment is metal independent but strictly related to the specific experimental conditions (^17^O water enrichment, temperature, and contact time).

## 5 Nature and Topology of Single Metal Binding Sites in Zeolites: The Role of 3d Orbitals

As outlined in the previous section, zeolites provide an ideal platform for the stabilization of single (transition) metal ions at sites, whose nature depends on the zeolite structure, Si/Al and metal/Al ratios. By controlling these parameters, single metal sites can be engineered prompting metal-loaded zeolites toward a number of important catalytic transformations.^43^ Copper- and vanadium-exchanged zeolites are relevant examples. In this case, the metal–oxide interaction involves the metal 3d orbitals, and ^17^O hfc measures the σ- and π-contribution to the chemical bond. During catalysis, Cu and V cycle through paramagnetic states with complementary electronic configurations, namely, Cu^2+^ (3d^9^) and V^4+^ (3d^1^). The siting of both elements over the large internal surface of the zeolite is primarily driven by electrostatic interactions brought about by the Al^3+^ distribution in the framework, while the fine details of the electronic structure arise from the favorable interactions between the metal 3d orbitals and the oxide frontier orbitals.

**Figure 4:**
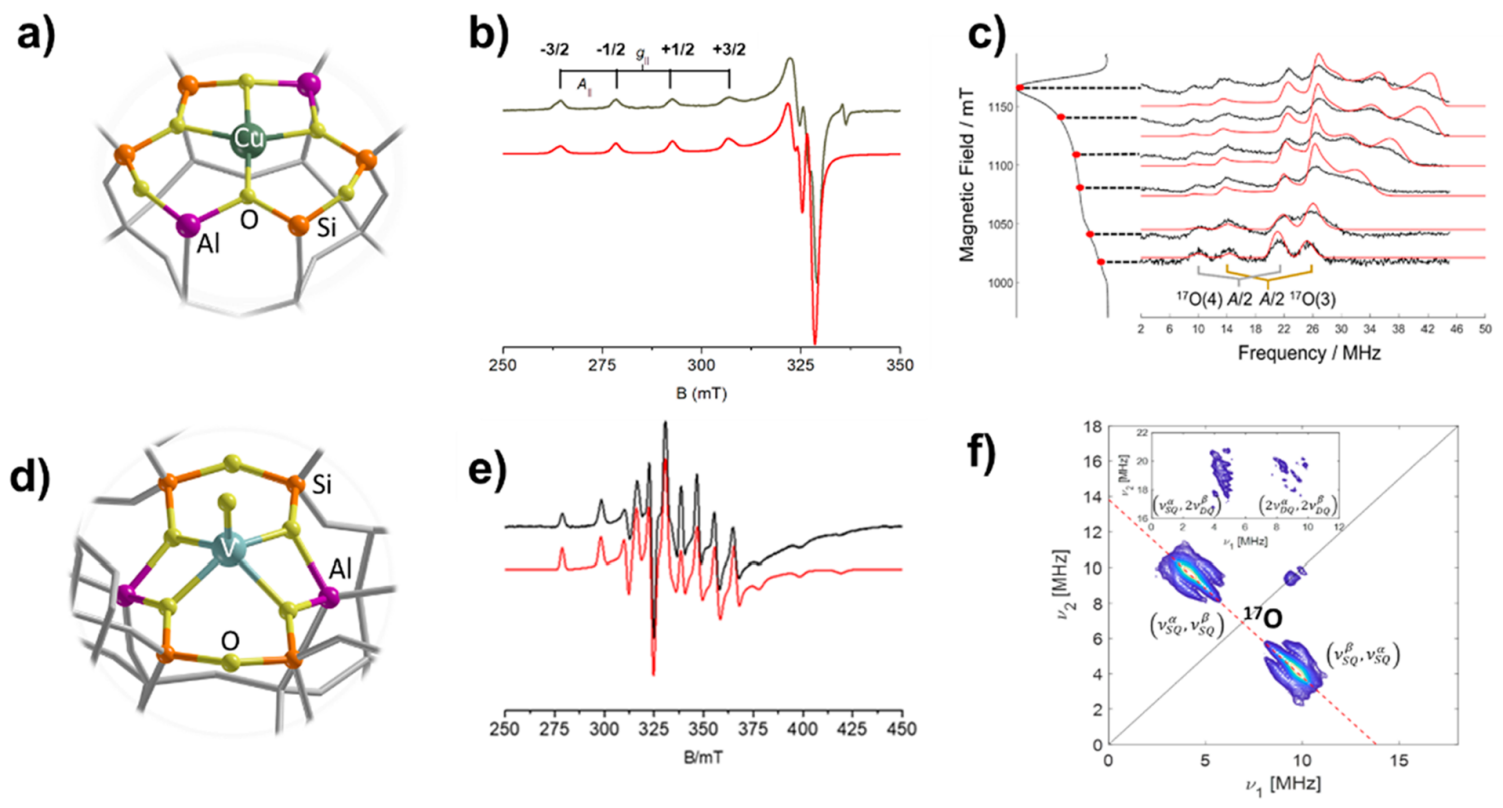
(a) Periodic model for single Cu atoms docked on CHA. (b) CW-EPR spectrum of single Cu ions on CHA. (c) ^17^O ENDOR spectra of single Cu ions on ^17^O enriched CHA. (d) Periodic model for single VO^2+^ ions docked on H-ZSM-5. (e) CW-EPR spectrum of single VO^2+^ ions on H-ZSM-5. (f) ^17^O HYSCORE spectrum of VO^2+^ ions on ^17^O enriched H-ZSM-5. The level of ^17^O enrichment at the metal site is estimated to be ≈70% in analogy with Zn/ZSM-5.^3^ Panels a, b, and c adapted with permission from ref (4). Copyright 2021 the Authors. Published by Springer Nature under the terms of the Creative Commons CC BY license (CC BY 4.0). Panels e and f reproduced with permission from ref (37). Copyright 2020 Elsevier.

Cu^2+^ species in dehydrated zeolites adopt an unsaturated square planar coordination in the proximity of two framework aluminum ions, that act as charge compensating agents (Figure 4a,b).^4,38^ Quantitative analysis indicate Cu^2+^/Cu ratio of 0.60 in the dehydrated material. The fine details of the metal coordination structure and the location of the Al in the framework play a crucial role in modulating the catalytic activity but are notoriously difficult to determine. We demonstrated that ^17^O EPR provides a robust handle for their quantification. ENDOR experiments performed on Cu-exchanged zeolites with Chabazite (CHA)^4^ and ZSM-5 (MFI)^38^ topology enriched in ^17^O show ^17^O coupling on the order of 60 MHz, similar to those observed in the case of Zn^+^ (Figure 4c) and characteristic of σ-bonding. Quantum chemical analysis of the spin density distribution shows that the ^17^O hfc is very sensitive to the local structural deformations induced by nearby framework Al ions, whose location can be pinpointed with accuracy.^4^

Similar siting sites stabilize V^4+^ species in ZSM-5 (Figure 4d), in the form of isolated vanadyl ions as revealed by CW-EPR (Figure 4e).^37^ However, ^17^O HYSCORE spectra (Figure 4f) detected only a minute (≈ 7 MHz) ^17^O hfc with framework oxide ions, which is nearly 1 order of magnitude smaller than the one recorded for Zn^+^ and Cu^2+^ in the same system. In this case the small ^17^O hfc is not related to a reduced covalent character with respect to Cu^2+^ and Zn^+^ but reflects the fact that the unpaired electron is localized in a nonbonding σ orbital and the measured ^17^O hfc weighs the degree of metal–oxygen π-bonding.

## 6 Single Metal Centers with 3d^1^ Electronic Configurations on the Surface of Reducible Oxides

**Figure 5:**
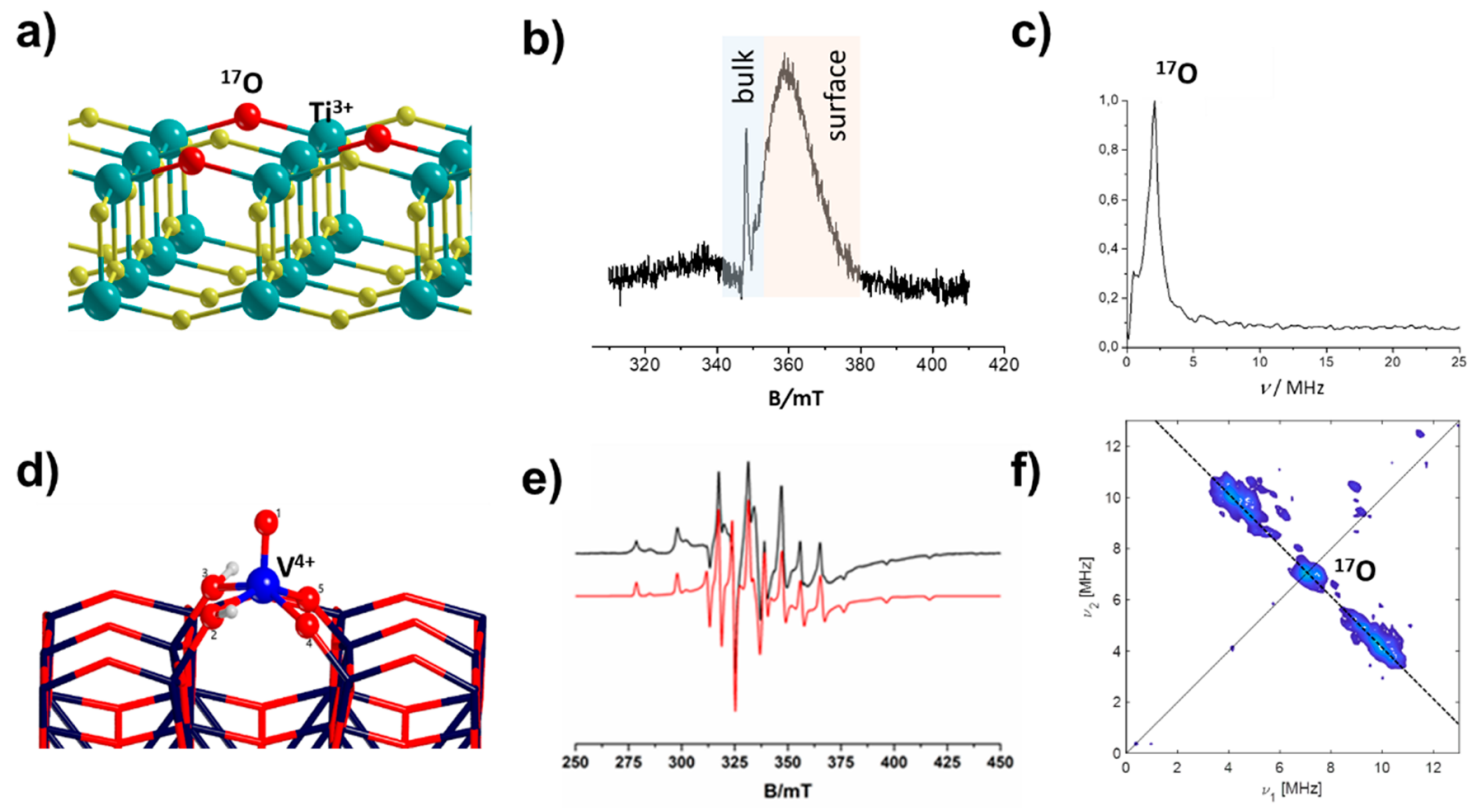
(a) Model of the (100) surface of TiO_2_ anatase. (b) Pulsed EPR spectrum and (c) ^17^O ESEEM spectrum of Ti^3+^ at the surface of anatase. (d) Model for single VO^2+^ ions adsorbed on the (100) surface of anatase. (e) CW-EPR spectrum surface (black experimental and red simulated spectrum) and (f) ^17^O HYSCORE spectrum of single VO^2+^ ions on ^17^O enriched on anatase (100). Panels a, b, and c adapted with permission from ref (2). Copyright 2011 American Chemical Society. Panels d, e, and f reproduced with permission from ref (32). Copyright 2022 Elsevier.

While alkali metal atoms bind on the surface of insulating oxides (MgO) through a polarization interaction (see section 4), on the surface of reducible oxides such as TiO_2_ they spontaneously ionize and forfeit any parentage in the electronic states of the gas-phase alkali atoms.^2^ The released “excess electrons” reduce Ti^4+^ forming paramagnetic Ti^3+^ ions, which can be stabilized either in the bulk or at the surface (Figure 5a). ^17^O EPR of TiO_2_ anatase selectively enriched at the surface provided evidence for surface localization of the Ti^3+^ species (Figure 5b,c).^2,51^ The small width of the ^17^O ESEEM signal is compatible with a maximum hfc of ≈3 MHz (Figure 5c). When dealing with solid-state semiconductors, it is instructive to benchmark the magnitude of the measured hfc against corresponding molecular complexes to gauge the extent of the wave function delocalization. For instance, the fully localized [Ti(H_2_^17^O)_6_]^3+^ yields a ^17^O hfc of 8 MHz,^58^ which is also similar to that measured for the strongly localized Ti^3+^ in the bulk of Ti^17^O_2_ rutile.^36^ Thus, comparison of the ^17^O hfc of oxygen atoms coordinated to Ti^3+^ species in different TiO_2_ polymorphs (anatase vs. rutile) together with the use of molecular complexes as a benchmark demonstrates that the electron wave function of Ti^3+^ at the anatase surface is characterized by a larger degree of delocalization. That excess electrons localize in rutile to form what in the physics’ trade is called a small polaron, while in anatase they prefer delocalized (free-carrier) states was independently confirmed by scanning tunneling microscopy and spectroscopy.^59^

The scenario is dramatically different when a transition metal ion with the same 3d^1^ electronic configuration such as V^4+^ is deposited on the surface of TiO_2_ (Figure 5d,e).^32^ In this case the observed ^17^O HYSCORE spectrum (Figure 5f) is characterized by cross-peaks separated by approximately 7 MHz corresponding to a hfc comparable to those observed in VO/ZSM5^37^ and reported for [VO(H_2_^17^O)_5_]^2+^ molecular complexes^25^ when the unpaired electron is fully localized. This comparison illustrates the profound difference in the electronic structure of an atom *at* the surface, i.e., part of the lattice (Ti^3+^, Figure 5a) versus an atom deposited *on* the surface (V^4+^, Figure 5d). The intrinsic differences between these two cases should always be considered carefully, when discussing the electronic structure of metals on oxides and surfaces in general.

## 7 Interfacial Coordination Chemistry of Single Metal Atoms and Ions

**Figure 6:**
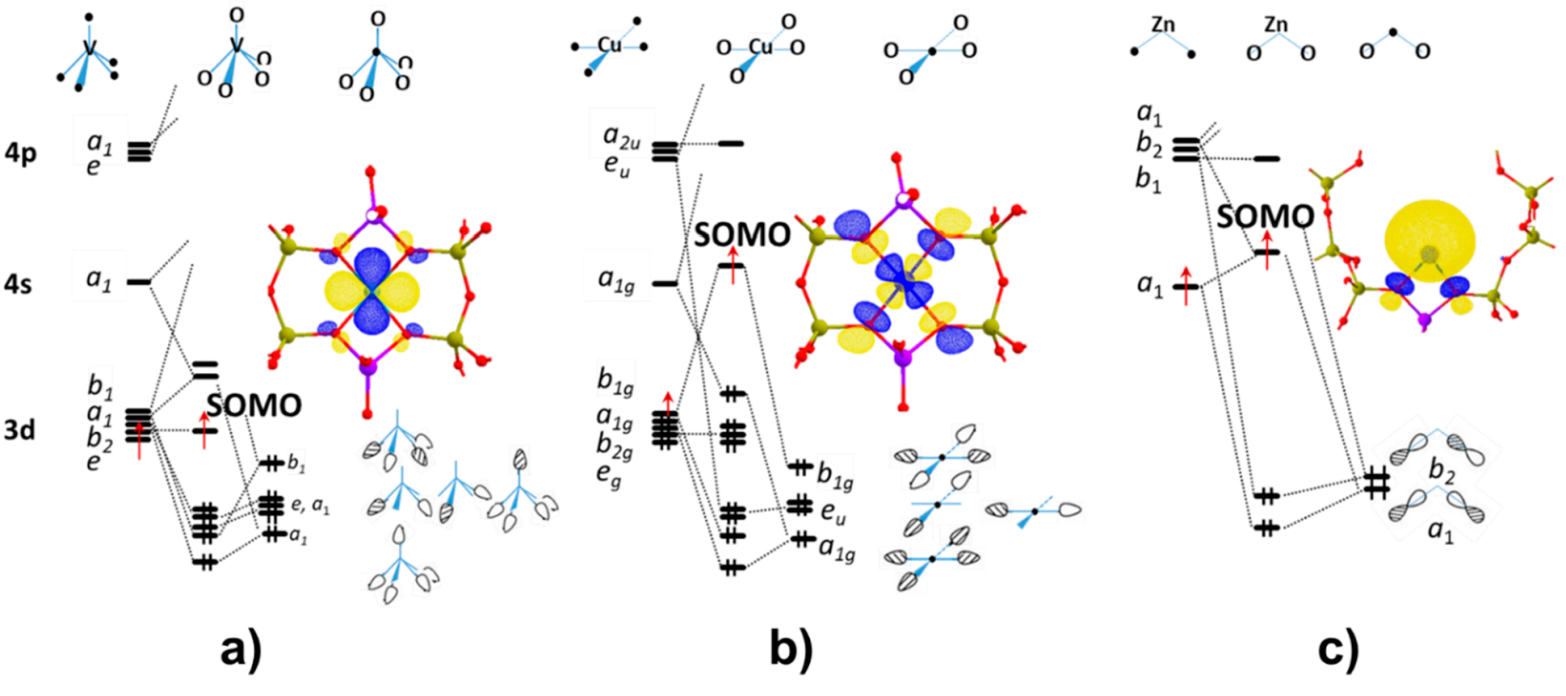
Simplified orbital correlation diagrams illustrating the sigma bonding of (a) VO^2+^ with ZSM-5 (diagram assumes *C*_4*v*_ local symmetry), (b) Cu^2+^ with ZSM-5 zeolite, assuming *D*_4*h*_ local symmetry, and (c) Zn^+^ on ZSM-5 assuming *C*_2*v*_ local symmetry. For each case, the SOMO was calculated on a cluster model at PBE0^60^/CP(PPP)^61^ level of theory (isovalue of 0.03).

The examples presented in the previous sections highlight the role of ^17^O as a particularly interesting target nucleus to study the metal–oxide interaction. To extract meaningful results, the measured hfc parameters need to be interpreted in the light of some knowledge of the electron wave function as obtained at different levels of accuracy in the frame of electronic structure theory.^8^ The use of simple interpretative models such as ligand field theory provides, however, a great deal of insight allowing us to establish sound correlations between classical coordination chemistry and the bonding of transition metal ions at surfaces. The properties of single paramagnetic atoms on oxide surfaces may be interpreted in a general way, considering that the unpaired valence electron does not materially affect the binding process but rather represents a convenient probe to measure the degree of interaction between the metal and the oxide adsorption site. A simplified orbital correlation diagram for the cases discussed in this Account, i.e., 3d^1^, 3d^9^ and 4s^1^ valence electron configurations, captures the essence of the problem (Figure 6). In the case of surface vanadyl ions (sections 5 and 6), the local geometry can be described in terms of a square pyramid with ideal *C*_4*v*_ symmetry (Figure 6a). Under these circumstances, the unpaired electron of the V^4+^ ion (*S* = 1/2, 3d^1^) is allocated in a vanadium 3d_*xy*_ orbital (singly occupied molecular orbital, SOMO) with nonbonding σ character and the weak π-interactions with framework O ligands are responsible for the small (≈7 MHz) ^17^O hfc. In the case of Cu^2+^ (*S* = 1/2, 3d^9^) the SOMO is the σ-antibonding orbital contained in the CuO_4_ square plane (Figure 6b), in which the Cu 3d_*x*^2^–*y*^2^_ orbital is combined out-of-phase with the 2p orbitals of O framework ligands. In this case the ^17^O hfc (≈60 MHz) is a direct measure of the degree of covalency in the Cu–O bond and crucially depends on the local structure distortion (bond lengths and angles).

In a similar fashion for the 4s^1^ elements (K^+^ and Zn^+^), the unpaired electron is allocated in a SOMO consisting of the out-of-phase combination of the metal 4s orbital and the 2p orbitals of coordinating O framework ligands (Figure 4c). In general, regardless of the atom considered, there is little electron transfer at the boundary between the metal and the surface of nonreducible oxides like MgO or zeolites (i.e., the unpaired electron is largely localized in the metal orbitals), but the fine details of the bonding interaction, crucially depend on the chemical nature of the substrates. In the case of the basic and ionic MgO, the metal–oxide interaction is dominated by strong polarization, while in the case of the isoelectronic Zn^+^ bound to the acidic and covalent ZSM-5 in a structurally similar surface site, the bond has a non-negligible covalent character, as revealed by the spin density sharing between the metal and the support. A completely different situation occurs for a reducible oxide such as TiO_2_. In this case neutral metal atoms (K) are fully ionized and the released electrons localize in the empty 3d orbitals of Ti^4+^ ions. The use of surface selective ^17^O enrichment allows us to monitor the fraction of electrons migrating to the bulk and those remaining at the surface. Moreover, the small ^17^O hfc indicates a significant delocalized character of the wave function of surface Ti^3+^ ions in anatase, a situation remarkably different from the rutile polymorph.

## 8 Concluding Remarks

Numerous experimental and theoretical studies aimed at a better understanding of the unique properties of single metal atoms and ions on oxide surfaces are currently being performed. Our studies demonstrate that ^17^O EPR can be a unique source of information on the structure and bonding interactions of open-shell single metal atom species. This is particularly important in cases of structural disorder often encountered in heterogeneous catalysts where the selective information from ^17^O EPR can be vital since other, more established structural techniques are not able to provide the same atomic-scale insight. We illustrated some of the guiding principles for a selective and atom-economic ^17^O isotopic enrichment of oxide surfaces and the application of EPR techniques to unravel fine details of the metal–oxide interaction. The chosen examples exemplify the role of the surface as a solid solvent whereby, depending on the oxide characteristics (acidity, basicity, reducibility), metal atoms or ions can be described in term of polarized atoms maintaining a recognizable parentage to gas phase species (alkali metals on MgO), coordination complexes (e.g., transition metal ions in zeolites), or ionized fragments (alkali metals on TiO_2_). The role of the metal support in modifying the electronic structure of single atoms on surfaces is highly system-dependent. The oxide support can affect the oxidation state of the adatom, stabilize unusual oxidation states and coordination geometries, or promote rearrangements of the orbital energy levels. Under all circumstances, the detection of ^17^O hfc is key to the understanding of the substrate-dependent changes in the electronic structure of supported single metal atoms on oxides, which are ultimately responsible for their unique catalytic properties. The level of structural information that can be obtained from ^17^O hfc and hyperfine techniques in general provides a powerful tool to help quantify and ultimately tune the multiple features required to design efficient single-metal catalysts, such as well-tailored electronic and geometric structure, high stability to sintering or leaching, and sufficient and uniform single site distributions. We expect this approach to be extended beyond the study of metal-oxide based catalysts to other substrates such as two-dimensional carbon-based hosts and other layered materials, where the most favorable coordination sites are usually provided by N, O, S, or P. Also, for this emerging class of materials, selective isotopic enrichment in conjunction with magnetic resonance techniques can provide unique opportunities to develop an atomistic understanding of the highly synergistic interactions between metals and supports that are the basis of unique chemical reactivity and catalytic performances of single-atom catalysts.
